# Australian black field crickets show changes in neural gene expression associated with socially-induced morphological, life-history, and behavioral plasticity

**DOI:** 10.1186/s12864-016-3119-y

**Published:** 2016-10-24

**Authors:** Michael M. Kasumovic, Zhiliang Chen, Marc R. Wilkins

**Affiliations:** 1 Ecology & Evolution Research Centre, School of Biological, Earth and Environmental Sciences, UNSW, Sydney, Australia; 2 Systems Biology Initiative, UNSW, Sydney, Australia; 3 School of Biotechnology and Biomolecular Sciences, UNSW, Sydney, Australia

**Keywords:** *Teleogryllus commodus*, Black field cricket, Developmental plasticity, Sexual selection, Gene expression, Transcriptome analysis, Behaviour

## Abstract

**Background:**

Ecological and evolutionary model organisms have provided extensive insight into the ecological triggers, adaptive benefits, and evolution of life-history driven developmental plasticity. Despite this, we still have a poor understanding of the underlying genetic changes that occur during shifts towards different developmental trajectories. The goal of this study is to determine whether we can identify underlying gene expression patterns that can describe the different life-history trajectories individuals follow in response to social cues of competition. To do this, we use the Australian black field cricket (*Teleogryllus commodus*), a species with sex-specific developmental trajectories moderated by the density and quality of calls heard during immaturity. In this study, we manipulated the social information males and females could hear by rearing individuals in either calling or silent treatments. We next used RNA-Seq to develop a reference transcriptome to study changes in brain gene expression at two points prior to sexual maturation.

**Results:**

We show accelerated development in both sexes when exposed to calling; changes were also seen in growth, lifespan, and reproductive effort. Functional relationships between genes and phenotypes were apparent from ontological enrichment analysis. We demonstrate that increased investment towards traits such as growth and reproductive effort were often associated with the expression of a greater number of genes with similar effect, thus providing a suite of candidate genes for future research in this and other invertebrate organisms.

**Conclusions:**

Our results provide interesting insight into the genomic underpinnings of developmental plasticity and highlight the potential of a genomic exploration of other evolutionary theories such as condition dependence and sex-specific developmental strategies.

**Electronic supplementary material:**

The online version of this article (doi:10.1186/s12864-016-3119-y) contains supplementary material, which is available to authorized users.

## Background

Developmental plasticity is common in continuously distributed phenotypes [1]. Although plasticity often leads to differences in morphological and/or behavioural traits (e.g., plasticity in response to pond drying; [2]), it is strongly linked to life-history traits driven by differences in development time [3]. It is specifically this life-history plasticity that is well studied both theoretically and empirically [1, 4] in ecological and evolutionary organisms. Decades of research on life-history driven plasticity has resulted in a strong understanding of the ecological triggers [5–8], adaptive benefits [9–11] and factors necessary for the evolution of such developmentally plastic tactics [12–14].

Despite the insight gained by studying life-history driven developmental plasticity in species with continuously distributed phenotypes, such species are often not ideal for the study of the role of genes in plasticity because it is difficult to assign continuous phenotypic differences to specific genetic variation. However, if the phenotypic consequences can be classified in a similar manner as to discrete morphs (e.g., horned beetles) [15] or life-history periods (e.g., hymenoptera) [16] it may allow for a clearer perspective on a gene-phenotype correlation [17] and provide insight into the underlying genomic control of developmental plasticity. In this study, we attempt to overcome this problem by using the Australian black field cricket (*Teleogryllus commodus*), a species that is well-described from an ecological and evolutionary perspective, and one where life-history driven plasticity can be categorized and followed through to maturity and death. We hope that exploring a species with a strong ecological understanding of the factors that result in continuous variation in phenotypic traits has the potential to highlight genes that may be important in life-history decisions and sex-specific variation in developmental strategies.

The Australian black field cricket is a well-studied organism with respect to life-history variation, mating strategies, and how selection affects each of these factors [18–22]. Additionally, both male and female *T. commodus* possess an interesting socially-induced developmental tactic [5]: males and females alter their resource investment and adult behavior depending on the density and rate of calls they hear in the last instar prior to maturity [23]. Males reared in an environment with a greater density of calls mature later and are heavier and larger than when reared under lower calling densities [23]. Males further match their own calling effort to their local competitive context [24], rendering them more competitive in a crowded environment [18]. In contrast, females in a high density environment mature smaller, but develop significantly faster, allowing them to exploit the high density of available males [23]. Females compensate for their smaller size by producing more eggs [23] and are able to make faster mating decisions [24]. This socially-induced developmental tactic [5] thus results in changes in the relationships between morphological, life-history and behavioural traits, associated with differences in development rate.

The aims of this study are: (a) to generate a de novo transcriptome for *T. commodus*, (b) to examine whether differences in nymphal gene expression can help explain the differences in developmental trajectories and adult behavior, and (c) to identify transcription factors relevant to the developmental trajectories. Identifying transcription factors in non-model organisms could provide particular insight into important pathways that align with specific life history tactics. One problem in identifying such transcription factors, however, is that they are often expressed in very low rates relative to other genes. We thus explored the expression of transcription factors using self-organised maps (SOM), which are a common bioinformatic technique used in *Drosophila* organ development [25, 26]. To do this, we reared males and females in two different simulated social environments and examined differences in neural genes expressed between the sexes, in two developmental environments, and in early and late stages of the last juvenile instar prior to maturity. To ensure that we could accurately match the adult morphological, behavioral, and life history traits to the genes expressed, we followed a large number of individuals after maturity until their death. Our results demonstrated that developmental differences correlated with changes in the expression of a small number of genes and transcription factors that regulate maturation, sexual development, and neural development. Moreover, the nymphal alterations in gene expression have lasting effects on adult behaviour and lifespan. We discuss these results with reference to the life-history and ecology of the Australian black field cricket.

## Methods

### Cricket rearing

Outbred recently captured wild type crickets were either 4th (genomics experiment) or 5th (rearing experiment) generation descendants of approximately 300 males and females collected at Smith’s Lake, NSW, Australia (32°22’S, 152°30’E). We collected nymphs before wing bud formation (which occurs at the penultimate juvenile instar). Each nymph was reared in an individual plastic container (5 × 5 × 3 cm^3^) with an egg carton for shelter and supplied with *ad libitum* food (Friskies Go-Cat senior) and water replaced weekly.

Upon molting to the last juvenile instar, we randomly assigned individuals to either a silent or a low density variable call-quality treatment. Although we have not yet examined the developmental tactic under silence, studies on *T. oceanicus* (a sister species) demonstrate that males moderate their mating strategies and sperm investment [27], while females moderate their mate preferences [28] in response to these environments. It is thus likely that these two extreme artificial rearing environments will have an effect in *T. commodus* as well. In the variable calling treatment, one of each of the three speakers (Logitech R-10) played a call from a different male at either the mean population calling rate (17 calls per minute), a high calling rate (24.5 calls per minute), or a low calling rate (12.6 calls per minute) [23]. We placed speakers in a one metre diameter circle and ensured that all speakers played calls at an amplitude of 70 dB at the centre of the circle. We reared individuals in two separate acoustically isolated environments and moved treatments between rooms each day to ensure no room effects.

For the genomics experiment, individuals were sacrificed and dissected at either 3 (early) or 13 days (late) after their last juvenile molt. We chose the early timepoint to allow for a comparison against the late timepoint, and also chose day 3 to minimize any gene expression differences due to molting to the penultimate juvenile instar. We chose the late timepoint because day 13 is the mean development time prior to maturation for crickets reared under 6 different artificial social environments [23]. Although individuals in the different treatments may be at different developmental stages when sacrificed, it allows us to explore the gene expression changes each individual experiences at the same time point. Furthermore, it would be extremely difficult to control for developmental stage in wild-type crickets. This allowed us to investigate whether gene expression differences exists between the two treatments at a point close to molting. We reared a total of 24 penultimate instar nymphs (12 male and 12 female) in two calling treatments (silent and low density-variable quality) and sacrificed individuals at two stages (early or late). This created a balanced design of three individuals (biological replicates) of each sex in each treatment in each time.

We reared another 701 crickets to sexual maturity as part of another larger experiment. For these individuals, we recorded their weight and size (pronotum width) at their final juvenile and adult instars within 24 h of molting into each instar. This allowed us to calculate their investment into adult size and weight while controlling for the initial starting value as [value at the juvenile instar – value at the adult instar] / value at the juvenile instar; we used these values in our statistical analyses below. After maturity, males were placed in an electronic recording device (callbox) to monitor their calling effort once a week [29]. Briefly, the callbox consists of 256 microphones attached to the lids of the housing containers which are connected to a data logger and personal computer. The computer is programmed to check for a signal from each microphone 10 times per second. The signal is recorded as 1 when 10 dB higher than the level of background noise, otherwise as 0. Calling effort is thus counted as the number of seconds a male is heard calling. Females were given a petri dish full of sand as a laying substrate to allow for the separation and counting of eggs.

### Statistics

We used a two-way ANOVA to examine whether there was a sex-specific effect of treatment on the investment into adult size and weight. We also examined whether there were any effects of treatment as a function of sex on development rate (days^−1^) and lifespan. We also examined whether there was an effect of treatment on adult reproductive effort, as average nightly calling effort in males and lifetime egg output in females using a GLM with a Poisson distribution and a log link. A proportion of individuals neither called nor produced eggs during their lifetime. Since there was no significant difference in the number of males (calling = 37, silent = 27; *χ* ^2^ = 1.18, P = 0.18) or females (calling = 19, silent = 23; *χ* ^2^ = 0.54, P = 0.46) that were not reproductively active between the treatments, we removed these individuals from our analyses.

### Dissections and extractions

We anesthetized Individuals on dry ice for two minutes prior to dissections. All brain dissections were performed in 0.01 M phosphate-buffered saline containing 3 % Triton X-100 on a bed of dry ice and completed within two minutes. We minimized temporal variation in gene expression by performing dissections between 1–2 pm each day. Upon completion of dissections, brains were immediately stored in a −80 °C freezer until extraction a maximum of 10 days later. We used a QIAGEN RNeasy Plus Universal Tissue Mini Kit for RNA extractions, following the manufacturer’s protocol.

### Library preparation and transcriptome sequencing

Brain tissue of 12 males and 12 females, equally from each rearing treatment (Silent, Calling) at two time points (Early: day 3, Late: day 13) were used for the isolation of mRNA using the Isolate II RNA Mini Kit (Bioline). The cDNA libraries for Illumina HiSeq 2000 sequencing were constructed from 10 μg of total RNA from each brain using the Illumina TruSeq RNA Sample Prep Kit (version 2) according to the manufacturer's instructions. Equal amounts of total RNA from each sample were barcoded separately (*n* = 24) after prep to allow for multiplexing in a single lane. Each lane containing the eight multiplexed libraries had an equal distribution of sexes, treatments, and timepoints to control for bias. Libraries were then sequenced on the HiSeq 2000 using TruSeq v3 SBS reagents to generate 101 bp paired-end reads with an approximate insert size of 160 bp, following the standard Illumina protocol. This resulted in an average of 80 million paired-end reads per individual. All sequencing was completed in the Ramaciotti Centre for Genomics, the University of New South Wales.

### De novo assembly of cricket transcriptome

Prior to RNA-Seq analysis, filters were applied to remove low quality reads from all twenty-four paired-end samples. Initial quality assessment for Illumina HiSeq sequence data was based on FastQC (version 0.11.2) (http://www.bioinformatics.babraham.ac.uk/projects/fastqc/) [30] statistics, and Cutadapt (version 1.2.1) [31] was used for adapter/primer trimming. We then trimmed paired-end raw reads with the BWA trimming mode at a threshold of Q13 (P = 0.05) as implemented by SolexaQA version 1.11 [32]. Low-quality 3’ ends of each read were filtered. Reads that were less than 25 bp in length were discarded.

RNA-Seq reads from 8 individuals containing each of the sex, treatment, and age conditions sequenced in the same Illumina lane were selected for assembly. This resulted in a total of 489.7 million 101 bp paired-end reads, that after trimming and filtering for quality and length respectively, gave 473.2 million PE reads. Transcriptome short reads were assembled de novo by ABySS then Trans-ABySS [33], Velvet-Oases [34] and Trinity [35]. The workflow for the transcriptome assembly, evaluation and annotation is summarised in Additional file 1: Figure S2 and greater details about the assembly and assemblers can be found in Additional file 2 : Supplementary Materials and Results.

The three assembled transcriptomes were compared by total size, N50 and sequence coverage. The Oases-assembled transcriptome had a total size of 199,904,425 bp made of 80,476 transcripts; this was the highest among the three assemblers. The Oases assembly also had the highest percentage of contigs covered by the other two assemblies (Additional file 1: Table S1). Reads from 24 individual samples were aligned to the three assemblies using Bowtie2 version 2–2.0.0-beta7 with the default parameters. The percentage alignment rates were calculated by Bowtie2 [36]. Manipulating alignment results involved the use of SAMtools version 0.1.18 [37]. The Oases assembly had the highest percentage of reads able to be mapped to the assembled transcriptome by Bowtie using default parameters; this was 0.5 % higher than the Trans-ABySS assembly, and 20 % higher than the Trinity assembly (Additional file 1: Table S1). Accordingly, the Oases-assembled transcriptome was selected as the candidate for further analyses.

We next evaluated the assembled transcriptome by comparing it to available reference transcriptomes to evaluate the quality of the de novo assembly results. The closest related species was the sister species, *T. oceanicus*. We obtained 41,962 de novo assembled *T. oceanicus* transcripts [38] and selected 32,643 transcripts of lengths greater than 200 bp for the comparison. A total of 50,945 *T. commodus* transcripts assembled by Oases had BLASTN hits to the 12,959 transcripts in the *T. oceanicus* transcriptome, of which 36,411 hits we re of high quality. We defined high quality hits as a minimum of 80 % alignment length of the pair of sequences and where the percentage identity is equal or greater than 80 % in the alignment as 80 % sequence similarity provides strong evidence of identifying two transcript sequences from similar species with similar function [39]. Among the high quality alignments, the average sequence similarity was 98.5 % and the average *T.oceanicus* transcript length coverage was 97.2 %. Although there is no gold standard for assessing transcriptome quality, these comparisons show that the assembled *T.commodus* transcriptome is at least comparable to the published *T.oceanicus* transcriptome. The *T.commodus* assembled transcriptome for all samples was submitted to the Transcriptome Shotgun Assembly (TSA) at NCBI; the details can be found at the end of the manuscript.

Despite the opportunity for comparison, the *T. oceanicus* transcriptome may not be a complete transcript set and not well annotated. We therefore decided to further validate our *T. commodus* transcriptome by performing a BLAST search against the complete set of transcripts of *Drosophila melanogaster* from FlyBase [40]. As the comparison is based on ortholog level, high quality hits were defined by a different rule as compared to the rule used in the *T. oceanicus* comparison. High quality hits were defined as a minimum of 80 % of the length of the reference sequence and a minimum of 50 % percentage identity in the alignment. A total of 47,763 hits to the *D. melanogaster* genome were identified by a BLAST search, of which 11,768 were high quality hits.

### Functional annotation and classification

To functionally annotate the cricket transcriptome, the final assembled transcripts (≥200 bp) were submitted for homology and annotation searches using Blast2GO software (version 2.4.4; https://www.blast2go.com/). For BLASTX against the NR database, the threshold was set to E-value ≤ 10^−6^. GO classification was achieved using WEGO software [41]. Enzyme codes were extracted and Kyoto Encyclopedia of Genes and Genomes (KEGG) [42] pathways were retrieved from the KEGG web server (http://www.genome.jp/kegg/).

Using BLAST2GO (version 2.4.4), we were able to assign gene annotations to 46,774 of the 80,476 transcripts from the Oases assembly. Gene ontologies (GOs) were also assigned to the assembled transcripts by BLAST2GO. There were a total of 90,357 gene ontology (GO) terms on all GO-levels associated with the 46,774 identified genes. Of these, assignments to level two GO-terms Molecular Function (40,244) made up the highest category, followed by Biological Process (33,225) and Cellular Components (16,888).

### Mapping of RNA-Seq and differential expression analysis

Gene expression levels were determined by quantifying the observed read abundance. As RNA-Seq reads can be mapped to multiple genes or isoforms, we used a read mapper capable of fully handling reads that map ambiguously between both isoforms and genes. We used the RNA-Seq by Expectation-Maximization (RSEM) package version 1.2.0 [43] with default settings to resolve ambiguous mappings and to perform final quantifications when assigning reads to genes and isoforms and counting transcript abundances. In each pair-wise comparison, we identified the significantly differentially expressed genes using the edgeR package [44], using the normalized read counts provided by RSEM. Although reference-based transcriptome analysis normally uses a q value < 0.1 and Benjamini and Hochberg FDR < 0.05, due to the nature of transcriptome assembly, the assembled transcriptome may have included a higher number of duplicated transcripts which would affect the read mapping and counting. Thus, to avoid missing any false negative values, we have chosen less stringent values for Benjamini and Hochberg FDR (10 %) and p value (0.05) for our analysis. We also provide a zip file with the expression values of the transcripts and their corresponding TSA transcript IDs to allow examination of the differentially expressed *T. commodus* genes in Additional file 3.

### Functional analysis of gene lists using DAVID

The Database for Annotation, Visualization and Integrated Discovery (DAVID) v6.7 is a set of web-based functional annotation tools [45]. The functional clustering tool was used to look for functional enrichment for corresponding *Drosophila* genes differentially-expressed in each condition. A unique list of gene symbols was uploaded via the web interface, and the background was selected as *Drosophila melanogaster*. We selected the Biological Process Gene Ontology as the functional annotation category for this analysis.

### Extraction of transcription factors

We downloaded a curated list of candidate *Drosophila* transcription factors identified on the basis of a structural domain assignment (for a DNA-binding domain) or previous Gene Ontology annotation for a transcription factor related term from the *Drosophila* Transcription Factor Database (v2.0) [46]. We used these transcription factor sequences as the queries for searching transcription factor sequences in our assembled cricket transcriptome. From the assembled transcriptome, 3,145 transcripts had BLAST hits to the *D. melanogaster* transcription factor list. For the transcription factor analysis, a final list of 2,418 transcripts from the *T. commodus* transcriptome was confirmed by excluding transcripts that had no read mapped in three or more individuals.

### Self-Organised Maps (SOMs) for extracting the expression pattern on Transcription Factors

The implementation of The Kohonen Self-Organizing Feature Map was used to build the SOMs. The average count of each extracted transcription factor from all 3 biological replicates of each condition is then calculated from the count matrix produced by RSEM and a new Average Count Matrix is build using these average counts and is used in the later steps. The average count matrix is then normalized by ‘genescale’ function in the ‘genefilter’ Bioconductor package [47] to have a mean of 0 and a standard deviation of 1. The Kohnonen pakage [48] in R then uses the normalized average count matrix to generate the SOMs. SOMs can be summarized in any number of grids, however, it is beneficial to choose a grid size that visually presents the gene expression patterns as clear separations in a distinguishable way. As a result, we trialed several different grid sizes and settled on a 5-by-5 grid as this provided the best visual separation of gene expression differences. The R scripts for generating the SOMs can be found at https://github.com/latrodektus/cricket_genomics.git.

## Results and Discussion

### Morphology, life-history, and behaviour

**Fig. 1:**
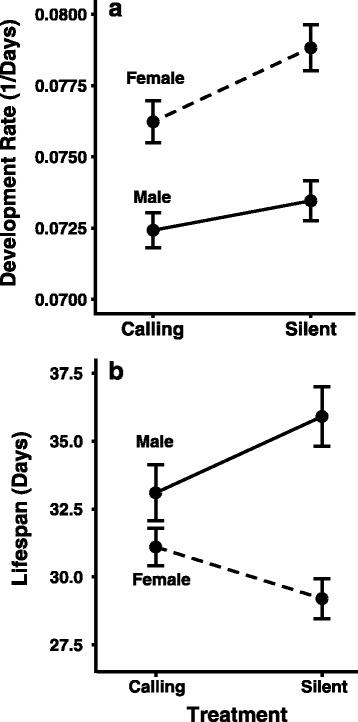
The difference in the developmental rate (**a**) and lifespan (**b**) of males and females reared in the silent and calling treatments. Bars are standard errors

Crickets were reared in two treatments; one was silent, the other where crickets were exposed to frequent, recorded calling. A total of 352 females (calling = 178, silent = 174) and 349 males (calling = 179, silent = 170) were approximately equally divided between the two treatments. As seen in our previous studies [23, 24, 49], there was a significant effect of treatment on the sex-specific expression of life-history, behavioural, and reproductive traits. Although there was no effect of treatment on either the investment towards body size or weight (Table 1), both males and females matured more quickly in the silent compared to the calling treatment (Table 1, Fig. 1). This treatment effect, however, is driven by females (Tukey HSD = 0.05) since there was no difference in the development rate between males in the two treatments (Tukey HSD = 0.74). There was also a difference in development between the sexes. Females developed faster than males (Table 1, Fig. 1), and also invested relatively more resources towards size (controlled for penultimate size; female: 0.156 ± 0.002 mm, male: 0.149 ± 0.002 mm) and weight (controlled for penultimate size; female: 0.665 ± 0.011 g, male: 0.567 ± 0.010 g) in their final instar relative to males. There was also a sex-specific effect of treatment on lifespan with males generally living longer than females; however, the silent treatment had the opposite effect on the sexes with males and females respectively showing an increase and decrease in lifespan (Table 1, Fig. 1).

**Table 1:** The effect of treatment and sex on four life-history traits

	F	d.f.	P
Size increase			
Sex	4.95	1, 697	0.03
Treatment	0.20	1, 697	0.65
Sex × Treatment	0.60	1, 697	0.44
Weight increase			
Sex	4.95	1, 697	0.02
Treatment	0.20	1, 697	0.65
Sex × Treatment	0.60	1, 697	0.44
Development rate			
Sex	41.13	1, 697	<0.0001
Treatment	6.46	1, 697	0.01
Sex × Treatment	1.19	1, 697	0.28
Lifespan			
Sex	22.55	1, 697	<0.0001
Treatment	0.24	1, 697	0.62
Sex × Treatment	6.76	1, 697	0.009

As a total of 30 males (calling = 18, silent = 12) died prior to being placed in the callbox, the analysis for calling effort is based on 319 individuals. The median nightly calling effort of adult males from the calling treatment was lower (1660 calls; 95 % CI: 3034–4193) than males from the silent treatment (2459 calls; 95 % CI: 2634–3968; *χ* ^2^ = 47.36, *P* < 0.0001). Adult females from the calling treatment had a higher median lifetime egg production (425 eggs; 95 % CI: 408–506) than females from the silent treatment (408 eggs; 95 % CI: 400–495; *χ* ^2^ = 47.36, *P* < 0.0001).

Our results thus replicate [23, 24, 49] and demonstrate four developmental and life-history tactics for which we can explore underlying differences in gene expression. First, as females invest more towards their growth and development rate relative to males (Fig. 1a), we expect to see a relative increased expression of genes associated with development, and maturation compared to males. Males, in contrast, invested significantly more resources towards lifespan (Fig. 1b) and we expect to see a relative increase in the expression of genes involved in life extension compared to females. Given that we also see a sex-specific effect of treatment on life-history and performance traits, we expect differences in sex-specific gene expression as a consequence of treatment. As males in the silent treatment had the longest lifespan and had a higher median-nightly calling effort, we expect a greater relative expression of genes associated with lifespan, energy metabolism, and courtship behavior compared to males in the calling treatment. In contrast, as females demonstrated a significant decrease in lifespan in the silent treatment compared to the calling treatment, accompanied with lower median reproductive effort, we expect a relative decrease in the expression of genes associated with lifespan and reproductive output in the silent treatment.

### Age-related gene expression differences

**Fig. 2:**
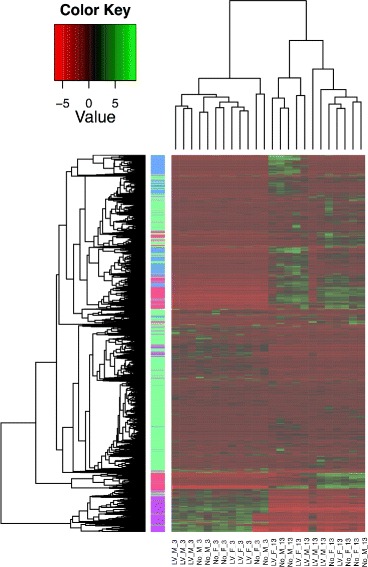
A heatmap showing the differential gene expression and clustering of samples according to expression values. The samples are on the left axis with notation for the treatments (LV = Low-Variable calling treatment; No = Silent Treatment), Sex (F = Female; M = Male), and age (3 = 3 Days; 13 = 13 Days). The top axis is the gene relationships, and the right axis is the clustering of the samples

To assess the gene expression profiles, sequenced reads from all 24 individuals were mapped to a de novo transcriptome, which we assembled with Oases (See Methods). We used RSEM version 1.2.0 with default settings [43] to assign reads to isoforms and to calculate transcript abundance. From all 24 samples, an average of 97 % of reads were mapped to the transcriptome by Bowtie. We provide a heatmap to show the differential gene expression and clustering of samples according to expression values in Fig. 2.

**Fig. 3:**
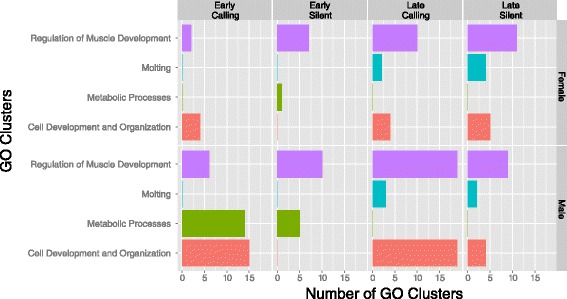
The number of gene ontology (GO) clusters demonstrated to be overexpressed by comparisons between each sex and each treatment within each time period. The GO clusters listed in the figure are GOs that are associated with the regulation of muscle development, molting, metabolic processes, cell development and organization, and molting in the early and late time periods. Each color represents a specific GO cluster. As comparisons are made within each time period, the bars do not represent relative differences in expression between sexes and treatments between time and thus cannot be compared between time periods

Differential gene expression analysis revealed significant differences in brain gene expression between crickets sacrificed on day 3 and those sacrificed on day 13 (>2-fold in expression and *p*- value <0.05). Due to the difficulty of analyzing the differential expression of a large number of transcripts (80,476 transcripts from 24 individuals) from both sexes between the two treatments at both time periods (2 × 2 × 2), we split the data into two temporal sets of transcripts (day 3 and day 13) as they contained related expression patterns (Additional file 1: Figure S1). In the temporally split sets, there were a total of 6,366 transcripts overexpressed in the brains of crickets sacrificed on day 3 compared to crickets sacrificed on day 13 (3,507 of which were successfully annotated), and 2,266 transcripts overexpressed in the brains of all crickets sacrificed on day 13 compared to all crickets sacrificed on day 3 (1,562 of which were successfully annotated). All the overexpressed transcripts in each time period fell into four Gene Ontology (GO) clusters: the regulation of muscle development, moulting, metabolic processes, and cell development and organization (Fig. 3, Additional file 2: Excel file).

In examining the temporal differences in expressed GO clusters, moulting-related genes accounted for the largest group of genes overexpressed in crickets sacrificed on day 13 compared to those sacrificed at day 3; 341 transcripts were significantly increased in their expression in the crickets sacrificed in day 13 compared to those sacrificed at day 3, while only 7 moulting related transcripts had significantly greater expression in crickets sacrificed on day 3 compared to those sacrificed on day 13. Crickets sacrificed on day 13 also had greater expression of 12 juvenile hormone esterase or epoxide hydrolase related proteins (proteins that trigger moulting, [50]) compared to those sacrificed on day 3. Only a total of 2 transcripts related to juvenile hormone epoxide hydrolase were found to be overexpressed in the early period, while 28 were found overexpressed in the later period. This difference between the crickets sacrificed on day 3 and 13 is explained by the synthesis, secretion, transport and accumulation of moulting proteins necessary to prepare for the moulting processes that occur closer to maturity. Our expression profiles described here thus seem to accurately describe the developmental progression from metabolic and catabolic processes required during early development, to the genes associated with maturation and moulting later in development.

We were initially surprised to see GO clusters associated with muscle development expressed in the brain, and it is likely that this expression is a result of the contamination from the muscle tissue surrounding the brain. Nonetheless, the pattern of increases in the GO clusters expressed in male crickets from the calling treatment sacrificed on day 13 is interesting as it follows the same pattern as those seen in metabolic processes, and cell development and organization GO clusters. This suggests that future studies focusing specifically on muscle may be interesting.

### Treatment and sex related differences

To delve more deeply into the expression differences between the sexes in each treatment for each time period, we mapped each of the transcripts to known *Drosophila* genes and focused on exploring the genes associated with biological processes in the behavioural, developmental, and life-history shifts demonstrated by juveniles in this study. As a result, we focused on genes that played roles in growth/maturation, lifespan, mating/courtship, flight/energy production, spermatogenesis/oogenesis, aggression, and memory/learning where the function is well documented by either mutant lines or knock-outs.

In day3, we mapped the 200–600 unique transcripts in each treatment by sex combination to 458 unique *Drosophila* genes. Of these genes, we found 25 genes that were overexpressed by one sex by treatment combination relative to the others, thus being unique to a single sex by treatment combination (Additional file 2: Excel file). In day13, we mapped the 200–600 unique transcripts in each treatment by sex combination to 563 unique *Drosophila* genes. Using the same procedure as in day 3, we found 21 genes unique to single sex by treatment combinations (Additional file 2 :Excel file). We discuss each of the treatment by sex combinations individually below and provide documented references and FlyBase IDs for each of the genes discussed below in the Additional file 2: Excel file.

#### Males reared in silence

**Fig. 4:**
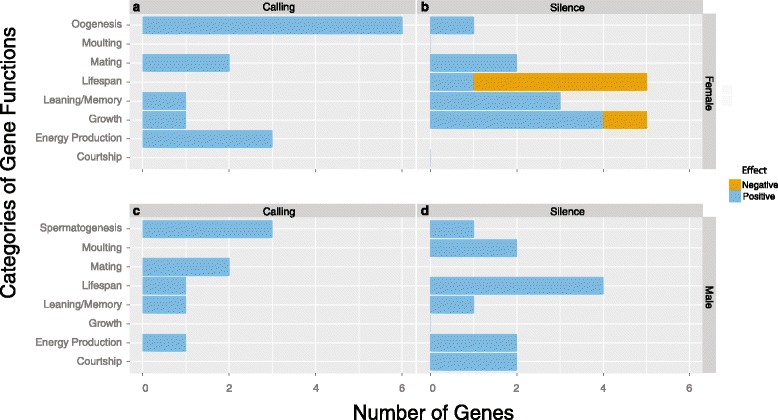
The genes expressed in the different sex (**a** and **b** vs. **c** and **d**) and calling treatments (**a** and **c** vs. **b** and **d**) in the different time periods. The red bars represent the number of genes expressed only in that functional group. The blue bars represent the number of genes in that functional group that are also expressed by different groups

Males in the silent treatment lived the longest (Fig. 1) and called the most. The significantly increased lifespan by males was paralleled with significantly higher expression of four separate genes associated with an increase in lifespan (Fig. 4). Males had higher expression of *ruby*, which directly contributes to increased lifespan [51]. Males also had higher expression of three genes that are known to indirectly positively affect lifespan: (1) *puckered*, which significantly increases lifespan through a decrease in reactive oxygen species [52, 53], increased immune system function [54], and wound healing [55, 56], (2) *p38b MAP kinase* which increases lifespan through immune response [57] and responses to ROS [58], and (3) *mitochondrial trifunctional protein α subunit* associated with increased lifespan through increased storage of lipid concentrations [59] and improved wound healing [60].

The increased calling effort shown by males in the silent treatment was paralleled by the expression of genes positively associated with courtship behavior and energy production, aspects that could result in more efficient calling effort (Fig. 4). All males reared in silence had a greater expression of *Neuroglian* compared to the other treatments. *Neruoglian* affects male courtship behavior with male *Drosophila* with increased expression performing a more intense courtship with higher courtship speeds [61]. This change in *Neuroglian* was paralleled with increases in genes associated with pathways of greater energy production. The first gene, *Glycerol 3 phosphate dehydrogenase*, is associated with changes in flight capacity of *Drosophila* due to increased tryglyceride energy stores [62]. The second, *Hyperkinetic*, is involved in potassium ion transport and regulates voltage-gated K channels in muscle fibers making them more efficient [63].

Even though males reared in silence did not mature more quickly than their counterparts reared with recorded calling, males from the silent treatment overexpressed two genes positively associated with moulting, *TATA box binding protein-related factor 2* which responds to ecdysone during the onset of moulting [64] and *ftz transcription factor 1* which is necessary for proper moulting through activation of ecdysone receptors by juvenile hormone [65]. It is interesting that these genes are expressed in males from the silent treatment as females from the silent treatment matured most quickly (Fig. 1A).

#### Females reared in silence

Females reared in silence demonstrated the most growth. Associated with their faster development, these females increased the expression of four genes whose expression is positively associated with growth and development (Fig. 4): (1) *bellwether*, a gene associated with the translation initiation factor *Eif4A* that behaves as a dose-dependent growth regulator [66], (2) *yorkie*, a gene involved in increased growth by positively regulating transcription [67, 68], and (3) *Juvenile hormone esterase* associated with increased growth [69] and mating [70]. Females also increased expression of *slimfast*, a gene where non-functioning mutants show growth similar to nutrient starved individuals [71].

Females reared in silence also demonstrated the shortest lifespan compared to all other animals (Fig. 1b). In line with this observation, of the five genes involved in lifespan expressed by females from the silent treatment (Fig. 4), four of the genes are negatively associated with lifespan. Specifically, increased expression of *myospheroid* directly results in a decreased lifespan [72] and *Autophagy-related 8a*, where suppression of this gene shows increased lifespan [73]. Overexpression of two other genes are known to decrease lifespan through a more indirect route: *superoxidase dismutase 2* is associated with reduced lifespan due to the costs of greater oxidative capacity [74] and *light* which interacts with the gene *blue cheese* which is associated with a decreased lifespan [51]. Females did, however express a single gene, *Neural Lazarillo*, where increased activity increases lifespan, but decreases growth [75].

The expression of genes associated with decreased lifespan is particularly interesting as they suggest a costly trade-off where females mature earlier, but live shorter lives. Studies specifically examining this trade-off through selection lines would provide a particularly interesting perspective on the association between development rate and longevity as these traits are shown to trade-off in other studies using *T. commodus* [76, 77].

Females from the silent treatment only expressed a single gene associated with egg output, *midway* [78], which may help explain the difference in egg output by females between the two treatments (see below).

#### Females reared with recorded calls

Females reared in the calling treatment only showed increased expression of a single gene associated with faster development, *myopic*, a gene that indirectly affects growth through its interaction with *yorkie* [79] (Fig. 4). This suggests that *yorkie* is an important factor in determining growth in *T. commodus* and may explain increased growth of females relative to males (Fig. 1).

Females reared in the calling treatment also produced relatively more eggs than females reared in the silent treatment. As was predicted, these females had higher expression of two unique genes, *spinster* and *tho2* each of whose expression is associated with increased oogenesis [80, 81]. Four other genes *Rab5* [82], *stumps* [83], *RNA-binding protein 9* [84], and *COP9 signalosome subunit 8* [85] are associated with germline maintenance necessary for proper oogenesis. Females from the calling treatment, however, also demonstrated increased expression of genes associated with energetic pathways (Fig. 4), increasing expression of *citrate synthase*, a marker of functioning mitochondria [86], *thiolase* which interacts with *mitochondrial trifunctional proteins* [59] and *NADH dehydrogenase (ubiquinone) 20 kDa subunit* which is involved in the electron transport chain [87].

Although not examined directly in this study, we discuss the gene expression results associated with mating and sexual communication in Additional file 2: Supplementary Materials and Results as these behaviors were examined in previous studies on a sister species, *T. oceanicus* [27, 28].

#### Males reared with recorded calls

In contrast to males in the silent treatment, males from the calling treatment expressed 6 unique genes, only one of which is positively associated with lifespan (*four wheel drive*) [88] (Fig. 4). This may explain the increased lifespan relative to females in both treatments, and the relatively decreased lifespan relative to males reared in the silent treatment (Fig. 1b). The other five genes were associated with mating and spermatogenesis (Fig. 4), behaviours that were not specifically examined in this study. However, because mating and spermatogenesis were not the focus of our study, but were examined in a sister-species, *T. oceanicus*, following a similar protocol [89], we discuss them in greater detail in Additional file 2: Supplemental Materials and Results.

#### Summary

Our above results highlight 45 candidate genes (Additional file 2: Excel file) that are associated with various life-history, morphological, and behavioural plasticity in our treatments and that have long been under study in *T. commodus* and other species. These results are intriguing for two reasons. First, we identified that the sex-specific differences in developmental strategies were associated with the differential expression of certain key genes, while the within-sex treatment differences were a result of the co-option of addition genes associated with the same developmental pathway. For example, females showed an increased expression of *yorkie* (a gene associated with increased growth) relative to males, while females reared in silence that grew larger varied in the expression of additional genes associated with increased growth. We found similar associations in patterns of lifespan; males reared in the silent treatment lived the longest and demonstrated the expression of four genes associated with increased lifespan, while females in the silent treatment had the shortest lifespan and expressed four genes associated with decreased lifespan. Males in the calling treatment demonstrated an intermediate lifespan and only expressed a single gene associated with increased lifespan.

Secondly, our results suggest that behavioural phenotypic outcomes are a result from associations of different genes interacting as modules. Whilst interaction of genes in modulating development is well known, our study confirms that this also occurs in an ecological, social and behavioural setting. For example, both males and females that increased their reproductive output had increases in genes associated with that trait and energy producing pathways. Males reared in silence called more, expressed a gene associated with greater courtship, and expressed two genes associated with the storage of greater energy reserves and the production of more efficient muscles. Females reared in the calling treatment produced more eggs, expressed six genes associated with germline maintenance and greater reproductive capacity, and expressed four genes associated with energy producing pathways. Our results thus suggest that the moderation of phenotypes in continuously varying species may be associated with the expression of additional genes, rather than dose-dependence of a smaller subset of genes. This, however, needs to be confirmed in future studies.

Because of the ecological and evolutionary understanding of the various phenotypes in *T. commodus*, we provide unique evidence for the focus of these genes in future evolutionary and ecological studies. Our results also allow researchers to further explore developmental tactics and the resulting phenotypes in other species from a genomic standpoint as we demonstrate similarities between the *T. commodus* and *Drosophila* genes, which likely extends to other species.

### Patterns of expression in transcription factors

Although transcription factors are not well explored outside of model genetic organisms such as *Drosophila*, identifying relationships between transcription factors and phenotypes in non-model organisms could provide particular insight into important pathways that align with specific life history tactics. To examine the expression patterns in transcription factors, we created a set of transcription factors containing 2,418 transcripts for *T. commodus* through comparison with the *D. melanogaster* transcription factor library (described in Methods). We used this set to explore differential expression of the transcription factors in each experimental condition. Similar to the differential expression analysis above, we used RSEM to create normalized count tables grouped by age (day 3 vs. day 13) due to the significant difference in gene ontologies used.

**Fig. 5:**
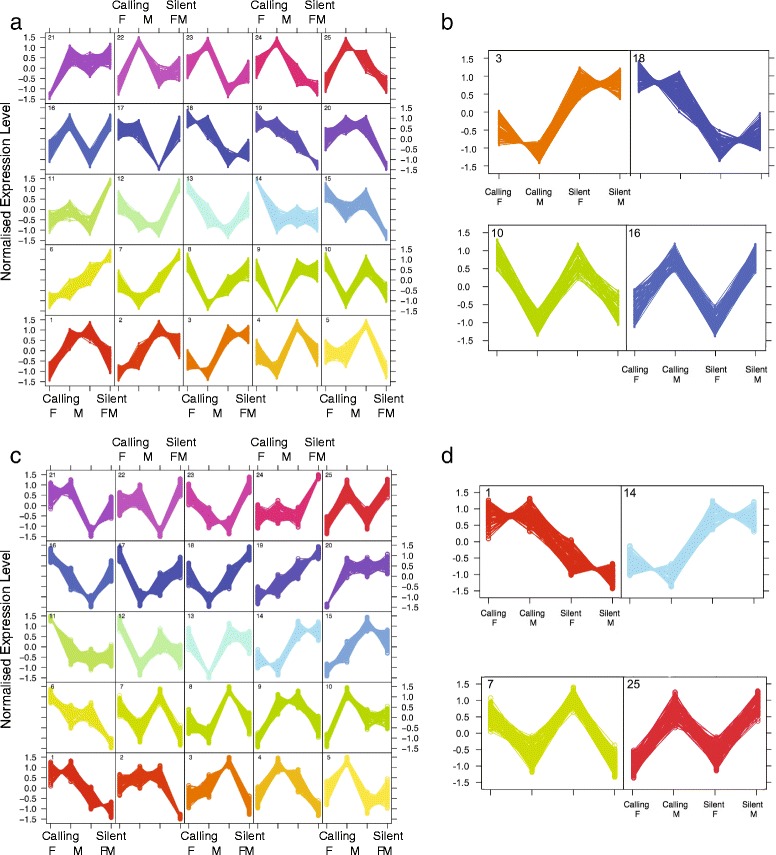
Self-organized maps (SOMs) showing the different expression patterns of the transcription factors expressed by individuals sacrificed at the two time periods: Day3 (**a**,**b**) and Day13 (**c**,**d**). In both figures, different expression patterns of the transcription factors in individuals of each age group were clustered into 5 × 5 grids by self-organized maps. The expression values are normalised to have a standard deviation of 1. Opposite expression patterns between treatments (Cells 3 and 18 in Early, Cell 1 and 14 in Late) and between sexes (Cells 10 and 16 in Early, Cell 7 and 25 in Late) are extracted and shown to the right of each 5 by 5 grid

Different expression patterns of the transcription factors in individuals of each age group were clustered into 5 × 5 grids by self-organized maps (SOMs; Fig. 5), as these best visually described gene clustering. For day 3, we next selected the two cells that had opposite expression patterns between treatments (Cells 3 and 18) and between sexes (Cells 10 and 16) (Fig. 5a). Cell 3 had a total of 126 transcription factors overexpressed in the silent treatment when compared to the calling treatment, while Cell 18 had 116 transcription factors overexpressed in the calling treatment when compared to the silent treatment. Cell 10 had a total of 89 transcription factors overexpressed in females when compared to males, while Cell 16 had 87 transcription factors overexpressed in males when compared to females.

We performed the same analysis for day 13 and found that Cell 14 had a total of 81 transcription factors overexpressed in the silent treatment when compared to the calling treatment, while Cell 1 had 93 transcription factors overexpressed in the calling treatment when compared to the silent treatment (Fig. 5b). In the sex comparison, Cell 7 had 92 transcription factors overexpressed in females when compared to males, and Cell 25 had 95 transcription factors overexpressed in males when compared to females.

We next performed a similar analysis as in the exploration of unique genes (above) to examine unique transcription factor expression, but limited our exploration to between treatments or sexes as the SOMs could not be created for a 2 × 2 × 2 interaction. We found a total of 31 transcription factors associated with the traits of interest in our study, 26 of which only appeared in a single sex or treatment. We discuss the two temporal periods together below.

#### Treatment differences in transcription factor expression

Individuals reared in the silent treatment had a slower development rate compared to individuals in the calling treatment (although this was driven by females; Fig. 1a). Despite this, individuals from the silent treatment expressed several transcription factors positively associated with growth and maturation, although each affected growth indirectly through interactions with juvenile hormone in some manner. *Foxo* is a transcription factor that regulates growth and the specific role it plays depends on the other genes that it interacts with [90]. *Ecdysone-induced protein 75B* is necessary for proper molting to occur [91], and *broad* is involved in ensuring proper expression of *let-7*, a small regulatory RNA that promotes transition from larva to adult [92]. *Ultraspiracle* is involved in tissue specific control of hormonal regulation [93].

Individuals from the calling treatment had a faster development rate, again largely driven by females (Fig. 1a). In contrast to individuals reared in the silent treatment, the two transcription factors directly play roles in development. The first, Topoisomerase 3α, is not only required for growth, but it is also involved in the more rapid growth necessary during compensatory growth [94]. The second transcription factor, *14-3-3ε*, acts as a modulator of *Foxo* [95] an important transcription factor that regulates growth [90].

#### Sex-differences in transcription factor expression

Females had a higher growth rate relative to males in our experiment. Females also showed an increased expression of Pdp-1, a transcription factor associated with growth [96]. Pdp-1 is also associated with increased deposition of fat, which may explain why females are heavier and may also be necessary for the energetic requirements of egg production. CG8578 is also associated with increased muscle development, but little is known about its actual function [97]. Females also demonstrated an overexpression of MTF-1, a transcription factor associated with increased lifespan as it maintains metal homeostasis [98]. Of the 4 transcription factors overexpressed by males, none seem to specifically relate to the traits studied here.

#### Summary

In our transcription factor analysis, we could not individually examine each sex within each treatment as in the overall gene expression results, thus resulting in weaker associations as transcription factors and the traits of interest. Nonetheless, we did see differences in the roles the genes played from simply being a part of the developmental process in individuals reared in the silent, to playing a regulating role in individuals reared in the calling treatment. Our results thus once again highlight the interactive role of genes in moderating individual development, and more importantly, place them within an ecological and social context. In each treatment or sex comparison, we found increased expression of several transcription factors that function in single or multiple functional processes. The fact that multiple transcription factors involved in similar roles increased in expression demonstrates redundancy within the developmental system, and could signify that genes are interacting with one another to result in an increased effect; this is similar to our genome wide transcriptome results above.

## Conclusions

The juvenile environment provides numerous cues regarding the potential challenges that individuals should encounter at maturity. If reliable enough [99], the presence of these cues should allow individuals to modify their investment patterns, thereby altering their developmental trajectory [1, 4, 5]. Despite having a strong understanding of the various ecological factors that trigger plastic developmental strategies, we have a poor understanding of the underlying genetic changes that accompany these developmental shifts. Are continuously distributed phenotypes a consequence of a dose-dependent reaction of particular genes? Or are phenotypic differences a consequence of the expression of additional genes with similar function? Understanding the underlying mechanistic patterns can help us understand the evolution of such plasticity, the extent of the potential constraints of plasticity, and the existence of sex-differences in developmental patterns.

Our study provides insight into each of these questions as we demonstrate that the socially-induced developmental plasticity of the Australian black field cricket (*T. commodus*) is associated with changes in the expression of suites of genes, including key transcription factors, associated with life-history, behavioural, and morphological traits that are under strong natural and sexual selection in this species. Additionally, because we looked at a specific subset of genes rather than simply gene ontology clustering, we provide numerous candidate genes and transcription factors whose roles were delineated through mutations and knock-outs using laboratory model species. Our results hint towards an association between gene function and phenotype as more extreme phenotypes were associated with the expression of a larger number of genes associated with that phenotype. This was seen in different trait domains as size, development time, egg output, courtship, and lifespan. Our results thus suggests that continuous phenotypes are a consequence of many interacting genes that together act as a dose-dependent regulator of a phenotype. Alternatively, the use of different genes may have the benefit of ensuring redundancy in developmental programs. Future studies examining whether developmental systems have finer control as a function of this redundancy and whether greater redundancy is common in species with continuous, rather than discrete phenotypes would provide greater insight into the evolution of plasticity.

We demonstrate that the different developmental tactics used by males and females in response to the same acoustic cues may be controlled by different subsets of genes, providing some insight to the sex-specific developmental strategies. For example, female *T. commodus* are larger and develop more quickly [100] and this is associated with the expression of a greater number of genes associated with larger size and faster development. Additionally, our lifespan differences between the sexes and the negative association between the genes expressed and lifespan specifically in females may be an example of the costs associated with particular developmental strategies. Although our study did not specifically examine sexual conflict in developmental strategies, further studies using the candidate genes outlined in this study to explicitly explore sex-specific trade-offs in development and behaviour as well as sexual conflict in mating [e.g., 20] and developmental strategies may prove fruitful.

Our results also show that in cases where expressing specific phenotypes is costly, such as increases in reproductive output, the genes associated with the trait of interest are coupled with increased expression in energy producing pathways. This suggests a second level of interaction between genes and the reliance between gene pathways. The relationship between such pathways may provide insight into questions of condition dependence [101, 102] – does increased investment in energy producing pathways reduce the cost of trait expression? Greater investment in underlying physiological systems may help animals to overcome certain costs of different life-history trajectories such that the costs are not readily apparent with the measure of a specific subset of phenotypic traits [103, 104]. This may make the identification of the costs of phenotypic plasticity more difficult to uncover [105, 106].

Our results are also surprising for a different reason. Despite finding strong developmental differences between the sexes and treatments, we did not find differential expression in some of the genes and pathways that are normally expected from such developmental shifts. For example, changes in the expression of heat shock proteins are often linked to increases in stress within the internal and external environment [107]. Relevant to our study, increased density is known to increase the expression of heat shock proteins, and this can subsequently increase longevity [108]. We saw no changes in heat shock proteins in our study, suggesting that auditory cues of density may not trigger the expression of heat shock proteins in the same way as physical cues of density. We also expected to see expression differences in the neuroendocrine axis [109, 50, 110] due to the size difference in females from the different treatments. We did not see a difference in dopamine expression, which is known to co-occur with juvenile hormone [111], and would therefore affect size at maturity. We also did not see expression differences in prothoracicotropic hormone (PTTH) which was surprising given that PTTH is secreted in the brain and begins the downstream expression of other hormones associated with molting. This, however, may be a consequence of the tight timing of PTTH with molting [109, 50] such that our sacrificing of individuals did not align with this timing. Alternatively, the differences associated with development in response to social cues may be cumulative compared to the relatively more abrupt physiological changes occurring during molting. Despite finding associations between lifespan and gene expression in our study, we did not see differences in the expression of methuselah, a gene that is strongly associated with lifespan in *Drosophila* [112]. This may be because although methuselah is associated with lifespan differences in the lab, such associations may not exist in wild populations despite natural variation in methuselah expression [113].

Overall, our results speak to the importance of examining expression patterns of ‘normal’ wild caught individuals as this demonstrates the interactive importance of genes in different phenotypic outcomes and that associations may vary between laboratory and natural populations due to the variation in selection. Our results also highlight the importance of examining the genomic underpinning of plasticity in response to various triggering factors as different environments and triggers may require the use of different developmental and physiological pathways. We also demonstrate that the genetic factors underlying developmental patterns can be uncovered in continuously variable species when a strong evolutionary and ecological understanding is coupled with a genomic approach [114]. Such an understanding cannot be gained through using laboratory strains of knock-outs and mutants alone [115]. This will hopefully encourage future genomic studies on non-model organisms.

## Additional files

Additional file 1: **Figure S1.** Expression patterns (log2-transformed, median centered) of the two clusters showing significant differences in gene expression between early (Day 3) and late (Day 13). The blue line indicates the mean-centered expression patterns of each cluster. The grey lines indicate individual expression patterns of each gene. **Figure S2.** Flowchart showing the workflow for the transcriptome assembly, evaluation and annotation. **Table S1.** The percentage of reads mapped to the three transcriptomes assembled by different assemblers. **Table S2.** Statistics of the assembled transcriptomes by different assemblers and redundancy removal steps. (DOCX 1765 kb)

Additional file 2: Supplementary Materials and Results. (DOCX 80 kb)

Additional file 3: Supplementary Excel file (XLSX 29 kb)
