# Supplementary material for: Australian black field crickets show changes in neural gene expression associated with socially-induced morphological, life-history, and behavioral plasticity
Source: BMC Genomics. 2016 Oct 24;17:827. doi: 10.1186/s12864-016-3119-y (PMC5078956; doi:10.1186/s12864-016-3119-y)
Supplement: Supplementary file 1 — Figure S1. Expression patterns (log2-transformed, median centered) of the two clusters showing significant differences in gene expression between early (Day 3) and late (Day 13). The blue line indicates the mean-centered expression patterns of each cluster. The grey lines indicate individual expression patterns of each gene. Figure S2. Flowchart showing the workflow for the transcriptome assembly, evaluation and annotation. Table S1. The percentage of reads mapped to the three transcriptomes assembled by different assemblers. Table S2. Statistics of the assembled transcriptomes by different assemblers and redundancy removal steps. (DOCX 1765 kb) [file 12864_2016_3119_MOESM1_ESM.docx]

Supplementary Figures

**S1 Figure 1. Expression patterns (log2-transformed, median centered) of the two clusters showing significant differences in gene expression between early (Day 3) and late (Day 13).** The blue line indicates the mean-centered expression patterns of each cluster. The grey lines indicate individual expression patterns of each gene.

**S2 Figure 2 Flowchart showing the workflow for the transcriptome assembly, evaluation and annotation.**


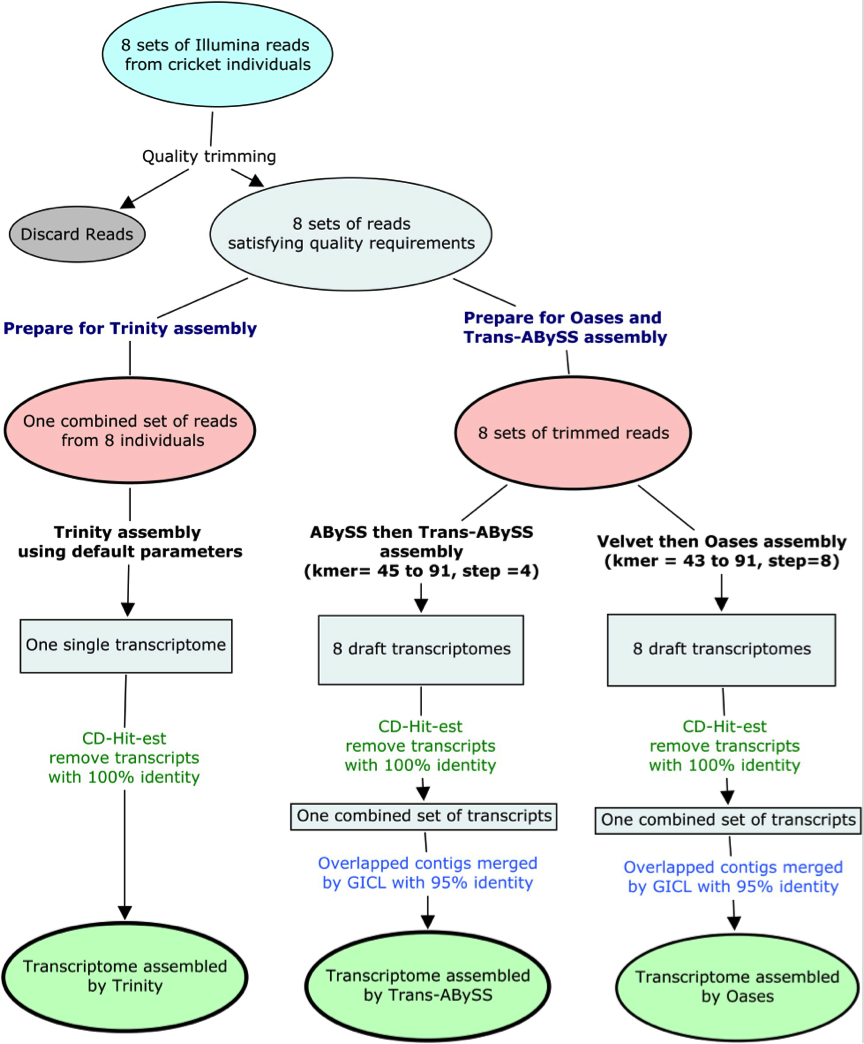


**S3 Table 1 The percentage of reads mapped to the three transcriptomes assembled by different assemblers.**

| Sample | Trans-ABySS (%) | Oases (%) | Trinity (%) |
| --- | --- | --- | --- |
| Calling_F_Day3_1 | 96.38 | 96.55 | 77.83 |
| Calling_F_Day13_1 | 96.50 | 97.18 | 81.00 |
| Calling_M_Day3_1 | 96.77 | 97.22 | 79.73 |
| Calling_M_Day13_1 | 96.61 | 97.21 | 80.65 |
| Silent_F_Day3_1 | 96.80 | 97.30 | 77.44 |
| Silent_F_Day13_1 | 96.34 | 96.99 | 80.54 |
| Silent_M_Day13_1 | 96.82 | 97.36 | 77.56 |
| Silent_M_Day3_1 | 96.07 | 96.58 | 82.60 |
| Calling_F_Day13_2 | 96.69 | 97.24 | 80.90 |
| Calling_F_Day3_2 | 96.13 | 96.56 | 81.75 |
| Calling_M_Day3_2 | 96.49 | 97.00 | 78.61 |
| Calling_M_Day13_2 | 95.56 | 96.01 | 82.11 |
| Silent_F_Day3_2 | 95.74 | 96.36 | 81.27 |
| Silent_F_Day13_2 | 96.44 | 96.81 | 78.95 |
| Silent_M_Day3_2 | 97.24 | 97.80 | 75.06 |
| Silent_M_Day13_2 | 95.68 | 96.11 | 80.40 |
| Calling_F_Day13_3 | 96.52 | 97.01 | 80.06 |
| Calling_F_Day3_3 | 95.92 | 96.54 | 80.42 |
| Calling_M_Day3_3 | 95.96 | 96.26 | 79.47 |
| Calling_M_Day13_3 | 96.20 | 96.80 | 76.81 |
| Silent_F_Day3_3 | 97.35 | 97.96 | 74.70 |
| Silent_F_Day13_3 | 95.97 | 96.57 | 80.61 |
| Silent_M_Day3_3 | 97.06 | 97.67 | 76.27 |
| Silent_M_Day13_3 | 95.81 | 96.34 | 81.76 |

**S4 Table 2 Statistics of the assembled transcriptomes by different assemblers and redundancy removal steps.**

|  |  | **Oases** | **Trinity** | **Trans-ABySS** |
| --- | --- | --- | --- | --- |
| **Original Assembly** | **Total # of contigs** | 558,712 | 87,741 | 1,473,008 |
|  | **Total Length** | 842,108,230 bp | 115,392,498 bp | 1,051,315,824 bp |
|  | **N50** | 2474 bp | 2,745 bp | 1636 bp |
|  | **Longest contig** | 19,564 bp | 28,092 bp | 16,448 bp |
| **After CDHit** | **Total # of contigs** | 378,707 | 87,065 | 701,150 |
|  | **Total Length** | 626,880,880 bp | 114,438,635 bp | 823,996,757 bp |
|  | **N50** | 2,691 bp | 2,749 bp | 1,901 bp |
|  | **Longest contig** | 19,564 bp | 28,092 bp | 16,448 bp |
| **After GICL** | **Final # of contigs** | 80,476 | N/A | 71,816 |
|  | **Total Length** | 199,904,425 bp | N/A | 108,183,727 bp |
|  | **N50** | 3,974 bp | N/A | 2,706 bp |
|  | **Longest contig** | 49,365 bp | N/A | 27,662 bp |
