# Supplementary material for: Australian black field crickets show changes in neural gene expression associated with socially-induced morphological, life-history, and behavioral plasticity
Source: BMC Genomics. 2016 Oct 24;17:827. doi: 10.1186/s12864-016-3119-y (PMC5078956; doi:10.1186/s12864-016-3119-y)
Supplement: Supplementary file 2 — Supplementary Materials and Results. (DOCX 80 kb) [file 12864_2016_3119_MOESM2_ESM.docx]

## Supplementary Materials

### De novo assembly of cricket transcriptome

Transcriptome short reads were assembled *de novo* by ABySS then Trans-ABySS ([Birol *et al.* 2009](#_ENREF_2)), Velvet-Oases ([Schulz *et al.* 2012](#_ENREF_46)) and Trinity ([Grabherr *et al.* 2011](#_ENREF_14)).

For the ABySS-Trans-ABySS and Velvet-Oases assembly strategies, we assembled datasets from each individual using similar assembly parameters (*k*mer value = 43 to 91 with step of 4). The Velvet-Oases assembly employed Velvet (version 1.2.07) using a set of *k*mer values ([Zerbino & Birney 2008](#_ENREF_61)), followed by Oases (version 0.2.08) with the default parameters ([Schulz *et al.* 2012](#_ENREF_46)). In ABySS-TransABySS assembly, individual *k*mer assemblies were carried out by ABySS version 1.3.4 with the scaffolding option off and contig end erosion off ([Birol *et al.* 2009](#_ENREF_2)). Trans-ABySS (version 1.3.2) was used after ABySS to merge the individual *k*mer assemblies with default parameters ([Robertson *et al.* 2010](#_ENREF_44)). The slightly different *k*mer set chosen for Velvet-Oases was due to its slow performance. However transcriptome assemblers do not simply group transcripts by individual assemblies from a single *k*mer, and instead gather all the transcripts and further assemble individual contigs into transcriptome. The chosen *k*mers for the three assemblers covers a very close range, and it has been proven that limiting the number of *k*mer values will not result in significant loss in assembly quality but will instead permit savings in assembly time ([Durai 2016](#_ENREF_11)).

After the initial assemblies, contigs of individual samples were merged with both strands information using the accurate mode of CD-HIT-EST version 4.5.4 with the sequence identity threshold at 100% and a word size of 8 ([Li & Godzik 2006](#_ENREF_27)). Since the combined set will contain small variations, such as allelic variations, small insertions or deletions, GICL (release date 2010-07-22) was then used to further reduce the redundancy level([Pertea *et al.* 2003](#_ENREF_40)). Contigs overlapped with at least 50 bp with a minimum identity of 95% were collapsed into single contigs, and the maximum length of unmatched overhangs was set to 100 bp.

Whereas for the Trinity assembly, we used a merged dataset from eight individuals. Trinity release 2012-06-08 was employed with the ALLPATHSLG error correction, and the paired fragment length was set to 200 bp. In the redundancy removal step, only CD-HIT-EST was used remove shorter transcript was entirely covered by longer one with 100% identity.

### Performances comparisons among Trans-ABySS, Oases and Trinity

There were no standard criteria to evaluate the quality of transcriptome assemblies ([Martin *et al.* 2010](#_ENREF_32)). Researchers usually assess the quality of an assembly mostly by looking at the contiguity and accuracy of the assembly ([Paszkiewicz & Studholme 2010](#_ENREF_38)). Here, we measured results in terms of transcript completeness, accuracy, and sample specificity, to compare the performance of three publicly available assemblers, Trans-ABySS, Oases and Trinity.

Trans-AbySS and Oases, the two multiple *k*mer assembly tools using an assembly merging stratage, outperformed Trinity, the single *k*mer assembler performing a single assembly with combined reads. In particular, Oases performed the best among the three assemblers, in assembly accuracy, contiguity and sample specificity. After the initial assembly, the Trinity assembly has the largest N50 and the least number of transcripts being assembled. The procedure of redundancy removal applied to the Trans-ABySS and Oases assemblies has greatly improved the quality of the transcriptome. In the final set of transcripts, the average contig length and N50 of the Oases assembly was significantly higher than those of Trans-ABySS and Trinity assemblies. Shown by the pairwise comparison, the Oases assembly overlapped more of their counterpart (Suppl Table 1). Oases’s highest proportion of transcripts being overlapped by other two assemblers also supports it as the best assembler in this study by transcript contiguity. Contiguity indices such as N50 can give an indication about how fragmented the recovered transcripts are.

RNA-Seq analyses often deal with multiple samples. The greatest concern for assembling samples individually is the increase of redundant transcripts, while assembling all samples at the initial step may result in the loss of sample-specific transcripts. We compared the sample specificity of different merging strategies in transcriptome assemblies in this study. For the Trans-ABySS and Oases assemblies merged from individual assemblies, in the initial stage they had more contigs that were much larger than those of Trinity. The merging of assemblies from individual samples using CD-Hit and GICL had greatly reduced the total number and size of transcripts and increase N50s (Suppl Table 2). The Trinity assembly based on merging all reads across all samples did not increase the proportion of mappable reads (Suppl Table 1). Although Trinity in principle provides additional information about isoform/paralog/allele structure of the transcriptome ([Grabherr *et al.* 2011](#_ENREF_14)), the low mapping percentage in sample specificity has shown that many of the isoforms assembled by Trinity may not truly reflect the real data.

Due to lack of genomic resources for the Australian black field cricket, the completeness of the transcriptomes was firstly measure by the BLAST search to an existing Hawaiian trigonidiine cricket gene index. Although the total number of hits from Oases assembly is slightly lower than that of from Trans-ABySS assembly, the number of high quality hits from Oases is higher. However, the completeness of the Hawaiian trigonidiine cricket gene index is remaining unknown, the number of hits to the Hawaiian trigonidiine cricket gene index cannot be used as an indication of transcriptome completeness, the *Drosopila* transcriptome from Flybase were considered as ‘gold standard’ reference in our studies. Among the three assemblers, Oases had the highest number of hits and high quality hits to the Drosophila transcriptome, it also had the highest number of high quality unique Drosophila transcript hits.

### Transcriptome Annotation

Gene name assignment is crucial for drawing biologically meaningful conclusions from RNA-seq experiments and for comparing results among different studies. Vijay and colleagues ([Vijay *et al.* 2013](#_ENREF_53)) suggested that in assigning orthologous genes from distantly related genomes, BLAST-based orthorlogy detection such as BLAST2GO would potentially have higher assignment success than suffix-tree based methods such as NUCmer and PROmer ([Kurtz *et al.* 2004](#_ENREF_25)). Stringent filtering on blast scores, alignment length and reciprocal-best-hits are thus crucial to guard against false detection of orthologous genes ([Chen *et al.* 2007](#_ENREF_6)).

To functionally annotate the cricket transcriptome, the final assembled transcripts (≥200 bp) were submitted for homology and annotation searches using Blast2GO software (version 2.4.4; http://www.blast2go.org/webcite). For BLASTX against the NR database, the threshold was set to E-value≤10^-6^. GO classification was achieved using WEGO software ([Ye *et al.* 2006](#_ENREF_60)). Enzyme codes were extracted and Kyoto Encyclopedia of Genes and Genomes (KEGG)([Kanehisa *et al.* 2004](#_ENREF_21)) pathways were retrieved from KEGG web server (<http://www.genome.jp/kegg/>).

Using BLAST2GO (version 2.4.4), we were able to assign gene annotations to 46,774 of the 80,476 transcripts from the Oases assembly. Gene ontologies (GOs) were also assigned to the assembled transcripts by BLAST2GO. There were a total of 90,357 gene ontology (GO) terms on all GO-levels associated with the 46,774 identified genes. Of these, assignments to level two GO-terms Molecular Function (40,244) made up the highest category, followed by Biological Process (33,225) and Cellular Components (16,888).

## Supplementary Results

### Gene ontology analysis

We also found that females reared in the calling treatment and males in the silent treatment overexpressed Osiris proteins compared to females in the silent treatment and males in the calling treatment, respectively. The Osiris gene family is a family of approximately 20 genes first described in *D. melanogaster* that are highly conserved and only found within insects ([Shah *et al.* 2012](#_ENREF_47)). Although the genes are still of unknown function, they are the molecular basis of the unique Triplo-lethal locus ([Lindsley *et al.* 1972](#_ENREF_29)) and have a secretion signal peptide and four domains, one of those being a putative transmembrane domain.

### Functional gene expression analysis

Although we did not study mating, spermatogenesis, CHC pheromonal communication, or learning in this study, these aspects have were the focus of previous studies in this and a sister-species, *T. oceanicus* ([Gray & Simmons 2013](#_ENREF_15)). As these studies followed a similar protocol, we discuss the genes associated with mating and oogenesis in greater detail here.

#### Males reared in silence

The results regarding spermatogenesis and mating behavior in our males reared in silence are unfortunately not as clear as our other gene-phenotype associations specifically examined in our experiment. For example, males in reared in silence also overexpressed single genes associated with mating success (*yellow*) ([Drapeau *et al.* 2006](#_ENREF_9)), and successful spermatogenesis (*Receptor for Activated C Kinase 1*) ([Kadrmas *et al.* 2007](#_ENREF_20)). Nonetheless, we mention then here as exploring these candidate genes in future research specifically examining sperm competitive ability and mating behavior may prove fruitful.

Females reared in silence

Females in the silent treatment only increase a single gene involved in mating decisions, *pale*, a gene associated with increased attractiveness between males and may also increase receptivity of females ([Liu *et al.* 2009](#_ENREF_30)).

#### Females reared with recorded calls

The calling treatment increased the expression of genes associated with mating and sexual communication. Females increased expression of *Desaturase 1*, which is associated with sexual communication through pheromones ([Houot *et al.* 2012](#_ENREF_17); [Marcillac *et al.* 2005](#_ENREF_31)), and along with increased oogenesis, *spinster* is also associated with increased mate receptivity ([Nakano *et al.* 2001](#_ENREF_36)). These results may help explain why females reared in higher densities of calls show increased receptivity and motivation to find males when searching ([Kasumovic *et al.* 2012](#_ENREF_22)).

#### Males reared with recorded calls

*Four wheel drive* is also associated with an increase in spermatogenesis during cytokinesis ([Brill *et al.* 2000](#_ENREF_3); [Giansanti *et al.* 2007](#_ENREF_13); [Polevoy *et al.* 2009](#_ENREF_42)) which may explain why *T. oceanicus* males reared in a calling environment demonstrate greater sperm viability ([Gray & Simmons 2013](#_ENREF_15)). In line with these increases in spermatogenesis, males also overexpressed *spargel*, a gene whose expression is associated with increased energy metabolism associated with mitochondrial regulation ([Rera *et al.* 2011](#_ENREF_43); [Tiefenböck *et al.* 2010](#_ENREF_52)). However, males reared in our calling treatment also demonstrated an increased expression of *Nascent polypeptide associated complex protein alpha subunit* ([Perrimon *et al.* 1996](#_ENREF_39)) and *Chromodomain-helicase-DNA-binding protein 1* ([Konev et al. 2007](#_ENREF_23); [McDaniel et al. 2008](#_ENREF_34)), genes associated with decreased fertility, in part due to decreased success in zygotic mitoses ([Konev *et al.* 2007](#_ENREF_23)); these gene expression results do not support the results from *T. oceanicus*. In addition, although males from the silent treatment had an increased expression of *lingerer*, a gene associated with increased copulation duration ([Kuniyoshi *et al.* 2002](#_ENREF_24)), males also had increased expression of *discs large 1* where mutants show decreased mating behaviour.

#### Learning and memory

In addition to the above, we also observed several unique genes involved in learning and memory expressed by males and females from the different treatments (Figure 3, Supplemental Excel file). Of these treatment by sex combinations, females reared in the silent treatment increased the expression of three different genes: *CRMP (*[*Morris et al. 2012*](#_ENREF_35)*)*, *Ankyrin 2 (*[*Iqbal et al. 2013*](#_ENREF_18)*)*, and *Argonaute-1* ([McCann *et al.* 2011](#_ENREF_33)) where disruption of the latter two results in cognitive impairment in learning and memory. Males in the silent treatment increased expression of *cAMP-dependent protein kinase 1* associated with memory increases ([Horiuchi *et al.* 2008](#_ENREF_16)) and learning ([Li *et al.* 1996](#_ENREF_28); [Skoulakis *et al.* 1993](#_ENREF_50)) and *Neuroglian* which is positively associated with neurogenesis ([Carhan *et al.* 2005](#_ENREF_4); [Yamamoto *et al.* 2006](#_ENREF_57)). In contrast, females in the calling treatment only expressed a single unique gene, *aru*, which is associated with memory formation ([Laferriere *et al.* 2011](#_ENREF_26)), and males in the calling treatment increased expression of *lethal (2)* *giant larvae*, which is involved in 26 different biological processes associated with neuronal and nervous system development.

Our results may help explain as to why individuals form other species that are reared under non-social conditions (in our case, in silence) can possess improved learning and memory retention ([Wongwitdecha & Marsden 1996](#_ENREF_55)). Future studies will be necessary to determine whether *T. commodus* reared in silence also have improved learning and memory.

**Transcription factors**

Here we outline the identified transcription factors that were not directly related to our phenotypic study, but yield interesting information for future studies in this and other organisms.

Individuals in the silent treatment

Two other transcription factors seem to play a greater role in males. The first, *spalt-related*, is associated with male genital development ([Si-Dong *et al.* 2003](#_ENREF_49)) and is also associated with antennal development and the sensory perception of sound ([Dong *et al.* 2002](#_ENREF_8)). Sound perception in *Drosophila*, however, is associated with antennal development, which is not the case in crickets. Thus, whether *spalt-related* has the same role in *T. commodus* is unknown. The second gene, *extra macrochaetae*, is associate with inter-male aggression ([Edwards *et al.* 2009](#_ENREF_12)), spermatid development ([Castrillon *et al.* 1993](#_ENREF_5)) and brain development ([Yamamoto *et al.* 2008](#_ENREF_56)).

Individuals from the calling treatment

Individuals from the calling treatment also showed an overexpression of transcription factors associated with reproduction: *daughterless* coordinates differentiation during follicle formation ([Smith *et al.* 2002](#_ENREF_51)), and *spindle E* is involved in 14 different roles associated with meiosis and oogenesis ([Zhang *et al.* 2000](#_ENREF_62)). These fall in line with our results that females from the calling treatment produced more eggs through their lifetime.

Expressed by females

Females expressed four unique transcription factors associated with neurogenesis when compared to males. *Domino* is a chromatin regulator that, among being associated with *E2F* (a key regulator of cell proliferation and differentiation, ([Parrish *et al.* 2006](#_ENREF_37)), it regulates dendrite development resulting in a greater number of longer branches ([Iyer *et al.* 2013](#_ENREF_19); [Ruhf *et al.* 2001](#_ENREF_45)). A second transcription factor, *cubitus interruptus*, is a component of hedgehog signaling and is essential for the development of dorsal class I da neurons ([Parrish *et al.* 2006](#_ENREF_37)). *Leonardo* (14-3-3ζ) is a gene that regulates protein folding and stabilizations ([Yano *et al.* 2006](#_ENREF_59)). In regards to our study, *Leonardo* is of particular interest as it is involved in facilitating olfactory learning and long-term memory in *Drosophila* ([Philip *et al.* 2001](#_ENREF_41)). These neuronal and sensory system transcription factors are likely to be particularly relevant for females as they are the mate-searching species.

Expressed by males

*neutralized* and *schnurri*, are associated with neurogenesis ([Yamamoto *et al.* 2008](#_ENREF_56)) and learning ([Dubnau *et al.* 2003](#_ENREF_10)), respectively. The other two transcription factors are not well documented; *PNUTS*, is associated with development and growth, however little is known about its exact function ([Ciurciu *et al.* 2013](#_ENREF_7)) and *female sterile*, is associated with gametogenesis in females ([Wieschaus *et al.* 1978](#_ENREF_54)).

Learning and Memory

We also found an overexpression of transcription factors associated with neuronal development. Interestingly there was a mixture of genes that positively and negatively regulate neuronal branching. There was an increase in expression of *14-3-3ε*, which, although involved in axon guidance, is not documented to play as central a role in neuronal development as *Leonardo* ([Yang & Terman 2012](#_ENREF_58)). Two other transcription factors negatively regulated neurogenesis; *Sin3A* functions in transcriptional repression ([Parrish *et al.* 2006](#_ENREF_37)) and *brain tumor* negatively regulates cell proliferation in brain development ([Bello *et al.* 2006](#_ENREF_1)) and neuromuscular junctions ([Shi *et al.* 2013](#_ENREF_48)). We also found an overexpression of three other transcription factors that are associated with neurogenesis according to FlyBase: *Without children, brahma*, and *daughterless;* however, their exact role in brain development is not well characterized.

**References**

Bello B, Reichert H, Hirth F (2006) The brain tumor gene negatively regulates neural progenitor cell proliferation in the larval central brain of *Drosophila*. *Development* **133**, 2639-2648.

Birol I, Jackman SD, Nielsen CB*, et al.* (2009) *De novo* transcriptome assembly with ABySS. *Bioinformatics* **25**, 2872-2877.

Brill JA, Hime GR, Scharer-Schuksz M, Fuller MT (2000) A phospholipid kinase regulates actin organization and intercellular bridge formation during germline cytokinesis. *Development* **127**, 3855-3864.

Carhan A, Allen F, Armstrong JD*, et al.* (2005) Female receptivity phenotype of icebox mutants caused by a mutation in the L1-type cell adhesion molecule neuroglian. *Genes Brain Behav* **4**, 449-465.

Castrillon DH, Gonczy P, Alexander S*, et al.* (1993) Toward a molecular genetic analysis of spermatogenesis in *Drosophila melanogaster*: characterization of male-sterile mutants generated by single P element mutagenesis. *Genetics* **135**, 489-505.

Chen F, Mackey AJ, Vermunt JK, Roos DS (2007) Assessing performance of orthology detection strategies applied to eukaryotic genomes. *PLoS One* **2**, e383.

Ciurciu A, Duncalf L, Jonchere V*, et al.* (2013) PNUTS/PP1 Regulates RNAPII-Mediated Gene Expression and Is Necessary for Developmental Growth. *PLoS Genetics* **9**, e1003885.

Dong PDS, Dicks JS, Panganiban G (2002) Distal-less and homothorax regulate multiple targets to pattern the *Drosophila* antenna. *Development* **129**, 1967-1974.

Drapeau MD, Cyran SA, Viering MM, Geyer PK, Long AD (2006) A cis-regulatory sequence within the yellow locus of Drosophila melanogaster required for normal male mating success. *Genetics* **172**, 1009-1030.

Dubnau J, Chiang AS, Grady L*, et al.* (2003) The staufen/pumilio pathway is involved in Drosophila long-term memory.

Durai DAS, M. H. (2016) Informed *k*mer selection for *de novo* transcriptome assembly. *Bioinformatics* **32**, 1670-1677.

Edwards AC, Zwarts L, Yamamoto A, Callaerts P, Mackay TF (2009) Mutations in many genes affect aggressive behavior in Drosophila melanogaster. *BMC Biology* **7**, 29.

Giansanti MG, Belloni G, Gatti M (2007) Rab11 is required for membrane trafficking and actomyosin ring constriction in meiotic cytokinesis of Drosophila males. *Molecular Biology of the Cell* **18**, 5034-5047.

Grabherr MG, Haas BJ, Yassour M*, et al.* (2011) Full-length transcriptome assembly from RNA-seq data without a reference genome. *Nat Biotechnol* **29**, 644-652.

Gray B, Simmons LW (2013) Acoustic cues alter perceived sperm competition risk in the field cricket Teleogryllus oceanicus. *Behavioral Ecology* **24**, 982-986.

Horiuchi J, Yamazaki D, Naganos S, Aigaki T, Saitoe M (2008) Protein kinase A inhibits a consolidated form of memory in Drosophila. *Proc Natl Acad Sci U S A* **105**, 20976-20981.

Houot B, Fraichard S, Greenspan RJ, Ferveur JF (2012) Genes Involved in Sex Pheromone Discrimination in Drosophila melanogaster and Their Background-Dependent Effect. *PLoS ONE* **7**, e30799.

Iqbal Z, Vandeweyer G, van der Voet M*, et al.* (2013) Homozygous and heterozygous disruptions of ANK3: at the crossroads of neurodevelopmental and psychiatric disorders. *Human Molecular Genetics* **22**, 1960-1970.

Iyer EP, Iyer SC, Sullivan L*, et al.* (2013) Functional genomic analyses of two morphologically distinct classes of Drosophila sensory neurons: post-mitotic roles of transcription factors in dendritic patterning. *PLoS ONE* **8**, e72434.

Kadrmas JL, Smith MA, Pronovost SM, Beckerle MC (2007) Characterization of RACK1 function in Drosophila development. *Developmental Dynamics* **236**, 2207-2215.

Kanehisa M, Goto S, Kawashima S, Okuno Y, Hattori M (2004) The KEGG resource for deciphering the genome. *Nucleic Acids Res* **32**, D277-280.

Kasumovic MM, Hall MD, Brooks R (2012) The juvenile social environment introduces variation in the choice and expression of sexually selected traits. *Ecology and Evolution* **2**, 1036-1047.

Konev AY, Tribus M, Park SY*, et al.* (2007) CHD1 motor protein is required for deposition of histone variant H3.3 into chromatin in vivo. *Science* **317**, 1087-1090.

Kuniyoshi H, Baba K, Ueda R*, et al.* (2002) lingerer, a Drosophila gene involved in initiation and termination of copulation, encodes a set of novel cytoplasmic proteins. *Genetics* **162**, 1775-1789.

Kurtz S, Phillippy A, Delcher AL*, et al.* (2004) Versatile and open software for comparing large genomes. *Genome Biol* **5**, R12.

Laferriere H, Ostrowski D, Guarnieri DJ, Zars T (2011) The arouser EPS8L3 Gene Is Critical for Normal Memory in Drosophila. *PLoS ONE* **6**, e22867.

Li W, Godzik A (2006) Cd-hit: a fast program for clustering and comparing large sets of protein or nucleotide sequences. *Bioinformatics* **22**, 1658-1659.

Li W, Tully T, Kalderon D (1996) Effects of a conditional Drosophila PKA mutant on olfactory learning and memory. *Learn Mem* **2**, 320-333.

Lindsley DL, Sandler L, Baker BS*, et al.* (1972) Segmental aneuploidy and the genetic gross structure of the Drosophila genome. *Genetics* **71**, 157-184.

Liu T, Dartevelle L, Yuan C*, et al.* (2009) Reduction of dopamine level enhances the attractiveness of male Drosophila to other males. *PLoS ONE* **4**, e4574.

Marcillac F, Grosjean Y, Ferveur JF (2005) A single mutation alters production and discrimination of Drosophila sex pheromones. *Proceedings. Biological sciences / The Royal Society.* **272**, 303-309.

Martin J, Bruno VM, Fang Z*, et al.* (2010) Rnnotator: an automated de novo transcriptome assembly pipeline from stranded RNA-Seq reads. *BMC Genomics* **11**, 663.

McCann C, Holohan EE, Das S*, et al.* (2011) The Ataxin-2 protein is required for microRNA function and synapse-specific long-term olfactory habituation. *Proc Natl Acad Sci U S A* **108**, E655-E662.

McDaniel IE, Lee JM, Berger MS, Hanagami CK, Armstrong JA (2008) Investigations of CHD1 function in transcription and development of Drosophila melanogaster. *Genetics* **178**, 583-587.

Morris DH, Dubnau J, Park JH, Rawls JM (2012) Divergent Functions Through Alternative Splicing: The Drosophila CRMP Gene in Pyrimidine Metabolism, Brain, and Behavior. *Genetics* **191**, 1227-1238.

Nakano Y, Fujitani K, Kurihara J*, et al.* (2001) Mutations in the novel membrane protein spinster interfere with programmed cell death and cause neural degeneration in Drosophila melanogaster. *Molecular and Cellular Biology* **21**, 3775-3788.

Parrish JZ, Kim MD, Jan LY, Jan YN (2006) Genome-wide analyses identify transcription factors required for proper morphogenesis of Drosophila sensory neuron dendrites. *Genes & Development* **20**, 820-835.

Paszkiewicz K, Studholme DJ (2010) De novo assembly of short sequence reads. *Brief Bioinform* **11**, 457-472.

Perrimon N, Lanjuin A, Arnold C, Noll E (1996) Zygotic lethal mutations with maternal effect phenotypes in Drosophila melanogaster. *Genetics* **144**, 1681-1692.

Pertea G, Huang X, Liang F*, et al.* (2003) TIGR Gene Indices clustering tools (TGICL): a software system for fast clustering of large EST datasets. *Bioinformatics* **19**, 651-652.

Philip N, Acevedo SF, Skoulakis EMC (2001) Conditional rescue of olfactory learning and memory defects in mutants of the 14-3-3 gene leonardo. *Journal of Neuroscience* **21**, 8417-8425.

Polevoy G, Wei HC, Wong R*, et al.* (2009) Dual roles for the Drosophila PI 4-kinase Four wheel drive in localizing Rab11 during cytokinesis. *Journal of Cell Biology* **187**, 847-858.

Rera M, Bahadorani S, Cho J*, et al.* (2011) Modulation of Longevity and Tissue Homeostasis by the Drosophila PGC-1 Homolog. *Cell Metabolism* **14**, 623-634.

Robertson G, Schein J, Chiu R*, et al.* (2010) De novo assembly and analysis of RNA-seq data. *Nat Methods* **7**, 909-912.

Ruhf ML, Braun A, Papoulas O*, et al.* (2001) The domino gene of *Drosophila* encodes novel members of the SWI2/SNF2 family of DNA-dependent ATPases, which contribute to the silencing of homeotic genes. *Development* **128**, 1429-1441.

Schulz MH, Zerbino DR, Vingron M, Birney E (2012) *Oases*: robust de novo RNA-seq assembly across the dynamic range of expression levels. *Bioinformatics* **28**, 1086-1092.

Shah N, Dorer DR, Moriyama EN, Christensen AC (2012) Evolution of a Large, Conserved, and Syntenic Gene Family in Insects. *G3: Genes|Genomes|Genetics* **2**, 313-319.

Shi W, Chen Y, Gan G*, et al.* (2013) Brain tumor regulates neuromuscular synapse growth and endocytosis in *Drosophila* by suppressing mad expression. *J. Neurosci.* **33**, 12352-12363.

Si-Dong PD, Todi SV, Eberl DF, Boekhoff-Falk G (2003) *Drosophila* spalt/spalt-related mutants exhibit Townes-Brocks syndrome phenotypes. *Proc Natl Acad Sci U S A* **100**, 10293-10298.

Skoulakis EMC, Kalderon D, Davis RL (1993) Preferential expression in mushroom bodies of the catalytic subunit of PKA and its role in learning and memory. *Abstracts of papers presented at the 1993 Cold Spring Harbor meeting on Neurobiology of Drosophila October 6-10, 1993.*, 200.

Smith JE, Cummings CA, Cronmiller C (2002) daughterless coordinates somatic cell proliferation, differentiation and germline cyst survival during follicle formation in *Drosophila*. *Development* **126**, 3255-3267.

Tiefenböck SK, Baltzer C, Egli NA, Frei C (2010) The Drosophila PGC-1 homologue Spargel coordinates mitochondrial activity to insulin signalling. *The EMBO Journal* **29**, 171-183.

Vijay N, Poelstra JW, Kunstner A, Wolf JB (2013) Challenges and strategies in transcriptome assembly and differential gene expression quantification. A comprehensive in silico assessment of RNA-seq experiments. *Mol Ecol* **22**, 620-634.

Wieschaus E, Marsh JL, Gehring WJ (1978) fs(1)K10, a germline-dependent female sterile mutation causing abnormal chorion morphology in *Drosophila melanogaster*. *Rouxs Arch. Dev. Biol.* **184**, 75-82.

Wongwitdecha N, Marsden CA (1996) Effects of social isolation rearing on learning in the Morris water maze. *Brain Res* **715**, 119-124.

Yamamoto A, Zwarts L, Callaerts P*, et al.* (2008) Neurogenetic networks for startle-induced locomotion in Drosophila melanogaster. *Proc Natl Acad Sci U S A* **105**, 12393-12398.

Yamamoto M, Ueda R, Takahashi K, Saigo K, Uemura T (2006) Control of axonal sprouting and dendrite branching by the Nrg-Ank complex at the neuron-glia interface. *Curr Biol* **16**, 1678-1683.

Yang T, Terman JR (2012) 14-3-3ε couples protein kinase A to semaphorin signaling and silences plexin RasGAP-mediated axonal repulsion. *Neuron* **74**, 108-121.

Yano M, Nakamuta S, Wu X, Okumura Y, Kido H (2006) A novel function of 14-3-3 protein: 14-3-3zeta is a heat-shock-related molecular chaperone that dissolves thermal-aggregated proteins. *Mol. Biol. Cell.* **17**, 4769-4779.

Ye J, Fang L, Zheng H*, et al.* (2006) WEGO: a web tool for plotting GO annotations. *Nucleic Acids Res* **34**, W293-297.

Zerbino DR, Birney E (2008) Velvet: algorithms for de novo short read assembly using de Bruijn graphs. *Genome Res* **18**, 821-829.

Zhang CX, Chen AD, Gettel NJ, Hsieh TS (2000) Essential functions of DNA topoisomerase I in Drosophila melanogaster. *Developmental Biology* **222**, 27-40.
